# An Empirical Study of the Operational Challenges for Private Animal Shelters in Hong Kong and Their Links to Regulatory and Policy Gaps

**DOI:** 10.3390/ani16192994

**Published:** 2026-09-23

**Authors:** Amanda Whitfort, Shuping Ho, Fiona Woodhouse, Marsha Chun

**Affiliations:** 1 Faculty of Law, The University of Hong Kong, Hong Kong, China; shuping.ho@spca.org.hk; 2 Society for the Prevention of Cruelty to Animals (Hong Kong), Hong Kong, China; fiona.woodhouse@spca.org.hk (F.W.); marsha.chun@spca.org.hk (M.C.)

**Keywords:** animal shelter, animal welfare, animal cruelty, animal rescue, unowned animals

## Abstract

We conducted a novel study of the current animal shelter landscape in Hong Kong. Seeking to identify the operational challenges faced by shelter owners and volunteers in meeting the needs of their animals, we conducted 22 interviews involving 10 large shelter operators and 12 long-term shelter managers/operations support staff. Eight interviewees had more than 20 years’ experience in operations. Interviewees identified inadequate regulatory oversight and lack of clear animal welfare policy leadership from the government as presenting serious impediments to the well-being of shelter animals. Interviewees reported that, in the absence of coherent and meaningful legislation and policies on animal welfare, they have become the primary bearer of responsibility for unsustainable numbers of unwanted animals and the authority on acceptable animal welfare practices, allowing for significant variations in standards. Our findings indicate that lack of government-led education setting out acceptable standards of care for animals and regulation of online fundraising is permitting those who seek to exploit animals for profit to use social media to ‘sell tragedy’ online, detracting funds from shelters already in financial need and undermining animal welfare in Hong Kong.

## Simple Summary

In 2025–6, we interviewed 22 animal shelter operators/long-term volunteers in Hong Kong to identify the primary obstacles they face in meeting the needs of the animals they care for. Interviewees reported that shelters are overwhelmed with unwanted animals and adequate funding to support their daily needs is difficult to secure. The major obstacles impeding the provision of adequate care for animals in shelters identified by interviewees were (a) lack of legislation setting minimum standards of welfare for animals, including those in shelters and (b) lack of regulatory oversight of online fundraising activities purporting to benefit unwanted animals. In the absence of minimum standards of care for animals and regulation of online donations, interviewees observed that social media posts of animals in need of urgent care were being widely used to ‘sell tragedy’, raising money from the public online without necessarily benefiting the animals concerned. We recommend the introduction of legislation setting minimum welfare standards for animals and oversight of online fundraising by the Hong Kong government to address the concerns identified.

## 1. Introduction

This study investigates the problems faced by shelter operators and their supporters seeking to improve the standards in animal shelters in Hong Kong today. The study is the first of its kind in Hong Kong and provides insights into stakeholders’ perceptions of the drivers for the problems they face. These insights are offered to assist policy makers in providing meaningful and legitimate support to shelters in improving the welfare of animals.

Despite only beginning to develop in earnest in the 2000s, private animal shelters play an important role in providing care for abandoned and unwanted animals in Hong Kong today. The earliest recorded shelter was provided by the Hong Kong Society for the Prevention of Cruelty to Animals (SPCA) which was founded in 1903 to campaign for the humane treatment of livestock and working animals [1] and opened its first dogs’ home in 1923.

The next local animal rehoming organisations, Lok Hong and the Society for Abandoned Animals (SAA), were established in 1993 and 1997 respectively. In 1997, the responsible department for animal management in Hong Kong, the Agriculture Fisheries and Conservation Department (AFCD), set up an Animal Welfare Advisory Group to advise the Director of AFCD on animal welfare issues including ‘the promotion of community awareness and responsible pet ownership’. However, no comprehensive animal welfare policy was articulated with regard to the management of unwanted animals at that time and those that were surrendered or collected by the Department were euthanized, if not claimed by an owner. While the SPCA already had this privilege, in 2003, Sally Anderson, the founder of Hong Kong Dog Rescue, set up an organisation to allow her take dogs from the government’s kennels for rehoming. These kennels are where unwanted dogs and cats were then, and continue to be, housed, after collection by the AFCD.

Since the late 1990s animal welfare organisations in Hong Kong have advocated for humane animal population management of unwanted dogs and cats through trap neuter return (TNR). To control population levels, TNR involves the trapping, neutering and return of animals to the original site from which they were collected. The SPCA set up a street cat TNR programme in 2000 which has since desexed more than 86,000 cats, reducing the numbers to manageable levels and dropping euthanasia rates for cats by 90% [2]. However TNR has been resisted by the AFCD for the management of unwanted dogs. Instead, the AFCD has relied on private shelters to take adoptable animals from its kennels for rehoming, with the remainder euthanized by the government.

The rehoming of animals was first mentioned in the AFCD’s 2009 Departmental report, showing 790 animals had been rehomed that year via rehoming organisations [3] who, following the example of the SPCA and HKDR, began to take animals from government kennels. In 2010, the AFCD’s Animal Management (Development) Division was established to implement outreach and education efforts regarding responsible pet ownership [4]. In 2011, an annual subvention of HK$1 million was set up to assist animal welfare organisations, including animal shelters [5]. The same year, the AFCD announced that it would cover the cost of desexing for animals adopted via animal shelters who had taken the animals from the government’s kennels [6].

The exact number of shelters in Hong Kong is unknown as current legislation does not require shelters to be licenced. However, a survey by students from the University of Hong Kong in 2025 found an online presence of approximately 50 shelters providing accommodation to dogs, cats, rabbits, reptiles and a small number of wild species. Many of these rely on fundraising from the public to support their operations. It is not necessary for shelters to register as charities to fundraise unless they seek donations from the public on the street and online fundraising is unregulated. Despite the growth of the government subvention support since 2011 to supplement donations, shelters report continuing to struggle financially.

## 2. Methodology

In 2025–6, we undertook a study of the current animal shelter landscape in Hong Kong. We sought to identify the operational challenges faced by shelter owners and volunteers in meeting the needs of their animals. We conducted 22 interviews involving 10 large shelter operators and 12 long-term managers/operations support staff in shelters. We initially identified interviewees through online searches for operating shelters and our professional connections. We contacted interviewees via written invitation to participate in the study and interviewed all responders. We asked all interviewees for further contacts, using the snowballing method to identify new participants. We continued to seek interviews until no new data was uncovered in the last 3 interviews. Interviews typically lasted 90–120 min. While specific subject areas pre-identified by the reviewers for the questionnaire were explored in each interview, open ended questions were used throughout the interview process. Some of the questions asked related to subject matter not described in this paper, such as welfare standards and sources of animals. All but one interview was conducted orally. The non-oral interviewee provided written answers to our questionnaire. The questionnaire used for all interviews is attached as Supplementary Material (Table S1: Shelter owner questionnaire) to this paper.

All interviews were conducted by the first or second author in English or Cantonese, respectively and recorded. The Cantonese interviews were transcribed into written English by the bilingual second author before analysis for themes. Participants were given anonymity to encourage candid responses to questions. Interviewees were offered the chance to read over the transcript of their interview before analysis to make any changes they wished. Shelter operators were offered a HK$200 supermarket voucher in compensation for their time.

A table setting out the number, gender and years of experience of our interviewees is provided below (Table 1).

After transcription, the 22 interviewee transcripts were analysed for re-occurring themes, with all responses on each theme tagged for further study. Overarching themes, each of which were flagged as repeatedly raised by interviewees, included attitudes to government, perceptions of charity regulatory oversight, issues around online fundraising, Trap Neuter Return programmes, euthanasia considerations, animal abandonment drivers, public attitudes to animals, social media impacts on animal shelters and volunteer behaviours.

Several key insights were identified concerning operational challenges and concerns for the long-term sustainability of shelters in Hong Kong. Most of these related to inadequate regulatory oversight and government support. These are presented in the five sub-sections in Section 3 below. Interviewees statements are provided in English (translated where necessary) to ensure transparency between the research findings and the views of the interviewees.

## 3. Findings

### 3.1. Funding Models

In another paper which arose out of this study we identified a common practice used by shelters in Hong Kong to raise funding. Many shelters use social media to fundraise. The majority of interviewees (13/22) complained that some shelters or animal rescue groups are regularly posting pictures of animals in apparent urgent need of medical care, providing quotes for vet bills alongside bank account numbers. Interviewees specifically labelled this form of fund raising ‘selling tragedy’, and complained that promoting urgency and crisis as a business model may successfully appeal to the emotions of supporters but is not amenable to long-term support of animal welfare, which requires consistent funding to support all animals in shelters, not just those in urgent need. Long-term shelters not relying on the crisis model complained that those ‘selling tragedy’ were undermining stable funding for day-to-day operations, in an environment where CSR funding is already difficult to obtain in the current poor economy. They complained that some shelters are keen to maintain the façade of desperation for money in order to attract supporters, keeping animals in a poor environment to assist in fundraising.

Shelter operators reported:

*Most people don’t pay attention; donations are very personal. People don’t care if it’s true or not, especially Hong Kong people. Time is very important. And they wouldn’t spend so much time investigating a thing, then go donate. It’s usually a story, the image is miserable. ‘If I have some spare money this month, I will donate it.’ This is very common, many of my friends are like this. Sometimes they will see a rescue. Those people put animals on Facebook: “I am raising money for medical bills now.” They don’t really search for what the group is. They feel that animal is miserable. They bank in one hundred HK dollars or two hundred HK dollars and think this is small money. But small money, seriously, has created another space for people to make money. I hear those stories.*

SS 05

*People donate money to you, hoping you will take the dog to a clinic. But they don’t care about the process. As long as your dog is cured, you take a picture and show it to others that the dog is recovered. They won’t go to the clinic to see your bill, how much money you spent, or where that money went. Does anyone know how much is embezzled? Nobody knows. I wouldn’t know.*

SO 04

*Many people raise money by ‘selling tragedy’ You will notice that we do not have these posts. Because we don’t want to emotionally blackmail people like that and not every animal is so pitiful. A tragic case may cost $20,000 but maybe the post raises $100,000. Where does the other $80,000 go? You will see some people in the animal protection industry not having to go to work, driving a luxury car. So I say some people do it initially because they like animals. Then later, they realise: I can make a living! So it’s not surprising they become full time animal rescuers.*

SO 06

*Having a charity, people think you must have people wanting to donate to you. People will look over us and think that we are actually doing OK. We are not pitiful enough because we really do not like to ‘sell tragedy’.*

SO 02

### 3.2. Requirements Around Registration as a Charity

In Hong Kong, bodies whose objective is the advancement of animal welfare have the option to register themselves as charitable institutions [7]. Registered charities are permitted to apply for tax exemption under section 88 of the Inland Revenue Ordinance, Cap 112, Laws of Hong Kong. Of the 10 shelter operators we interviewed, 8 had registered as charities and applied for tax exemption and one was waiting on the government to approve the application. Without registration as charities, some shelter operators reported that they were less likely to receive donations due to the public’s confidence being undermined by their inability to monitor how their donations are spent through published government records [8]. However applying for the licence was noted by 6 interviewees to be difficult and time consuming for animal welfare organisations without administrative support. Interviewees complained that with no legal requirement to register as a charity to do online fundraising, the benefits in becoming a charity were less tangible than they should be.

Charities in Hong Kong must file their accounts with the Inland Revenue Department and online. However problems with oversight of charities were widely identified. A number of interviewees complained that a charity which had been convicted of animal cruelty had been permitted to maintain its charity status registration. Another operator, convicted of serious animal-related offences, had opened a new shelter under an invalid charity licence number and raised funds online without repercussions. One shelter operator described an attempt by others to buy their charity licence as well as being told by their auditor that the Inland Revenue Department was on the inquiry as many NGOs were laundering money. The auditor was reported to have told the interviewee that money laundering through charities was a known issue amongst accountants who audit charity accounts.

Some interviewees were of the view that registration as charities should not be mandatory:

*Everyone is fundraising now. It’s not like only if I have a charity license can I raise funds. For example, if Mr. A or Mr. B finds a cat on the street and takes it to an animal clinic, they can raise funds themselves. Currently, many practices involve so-called independent volunteers. Some may be more experienced, while others may be completely new. They don’t mind using their own space to take a cat home and take it to the vet. Afterward, they look online to see if there are any kind-hearted individuals willing to donate to help cover the medical expenses. I think this is not a bad option. If everything is regulated and only registered charities can help, then your ordinary volunteers/public will have much less flexibility.*

SS 01

*I think online donations should be regulated. Actually, many organizations aren’t registered under section 88, so their donation accounts are likely private. If someone donates to a personal account, they won’t receive a tax-free receipt. However, they can also voluntarily donate to anyone. Well, it’s not exactly a donation, it’s a fund transfer. But my point of view is that we shouldn’t say people can only donate to those registered under section 88. I think it’s a personal choice. But it would be helpful to have a list of organisations that have the 88 license for transparency.*

SS 06

Some observed that holding a charity licence undermines their ability to fundraise:

*In terms of fundraising I don’t think there is much benefit in being registered as a charity. Or maybe there is not enough education for the general public because some people will think you’re a charity because you can raise funds.*

SO 06

*Because I hold a charity license people assume I have regular donors. This is already a misconception.*

SO 02

Transparency and oversight were flagged as particular problems by 12 interviewees.

*AFCD currently has a website that has a list of animal welfare organisations with licences the government supports. I don’t think that’s enough. Perhaps the list should include the organisations’ previous year’s annual report, if any, to let the public know where the money was spent and what percentage it was. Because we have an audit, we need to let the public know how much is spent on medical treatment and how much staff is paid. Perhaps there should be transparency to let the public know so they can choose which one to go to.*

SS 06

*Some shelters use the dogs to appeal for money, may be more than a million is asked for and they only need a few thousand but we don’t know where the money goes. They should have to be registered to receive donations. Some of the shelter owners pay themselves a high salary and take their personal expenses from the shelter income. I saw some ad for animals that ask for more than a few hundred thousand to do some vet care. There should be transparency in showing what the care actually cost. They never say how much they got and what they did with the balance.*

SS 12

*So you think this audit report is where you can see the percentages. The most important thing is you can see the distribution. But actually, regarding the content, you might not know if it’s true or false. The content they report might be fake, I think. Because veterinary clinics need to do business, they have to comply with the volunteers’ requests, which is equivalent to commissions. They also offer discounts of 20% and 30% on orders. All of this comes from the volunteers’ commissions*.

SS 02

*I know at one time, many shelters were laundering money. The auditors told us. I have myself received a message asking to acquire our s88 licence. The auditor explained to me: ‘A sum of money comes in, you can’t ask why because there is no source, it is an anonymous donation. A mysterious amount of cash deposited into your bank account. When you take the money out to pay the vet, it is washed clean.’*

SO 05

### 3.3. Fraud

While seeking donations on the street requires a permit from the Social Welfare Department and a charity registration to be legal, Hong Kong has no legislation to control online fundraising. In the absence of regulation of fundraising and mandatory auditing of accounts, the majority of interviewees described emotive posts used to secure funding for animals and 6 raised questions of fraud.

*Some are black-hearted, really looking for animals to make money. I think now the new batch is all doing that. I think these volunteers have come about in the last 10 years. They are very young, in their thirties. They are focused on asking for money. If you donate to some private individual, you don’t know if they’ll use the money to buy an iPhone 16, or maybe they’ll buy a sports car.*

SS 02

*If I didn’t accept donations, I could do whatever I wanted; I wouldn’t need to explain myself to anyone. Since I accepted donations, I really need to explain to those who donated to us what I was doing. I often feel that photos are very deceptive. people are so naive. They open social media and think, “This organization is so kind!” They go and feed the dogs and cats. But it’s not like that. The photos are easy to fake. I know some people around me who have an injured animal that needs to be taken to a clinic. They received donations from others, but instead of going to a proper clinic, they went to some black-market vets or even treated the animal themselves*.

SO 04

*I’ve also heard volunteers say they donated to XXX shelter and they might have donated a few pallets of food, but then the shelter said they ran out of food shortly after that. The volunteer felt there was no reason [since they just donated some]. What happened? Did the dogs really eat all the food so quickly? Or did the shelter distribute/donate it to other people? Or did they really pocket the money?*

SS 06

While shelters need not have a licence in Hong Kong, those that offer boarding, even overnight to an animal, for a fee, must be registered as a business and are subject to monthly inspections and licencing conditions on space, ventilation, lighting, and sanitation along with licencing and vaccination requirements for animals being boarded. Some shelter operators complained that the lack of regulation on shelters was being utilised to operate illegal businesses.

*Unfortunately there are bogus “shelters” set up with government grants and on government land that are just fronts, running businesses behind the facade.*

SO 09

*If they are lying, it can be found out, if you really check by looking at their accounts and by doing undercover operations. You can ask “I want to put my dog here for 10 days because I am going on holiday. How much is it?” If they give you a price, then they are not a charity. You can also tell by looking at the dogs. Our dogs are all rather bedraggled; if the dogs are all beautiful [or pedigree], then you know that they are doing boarding.*

SO 03

### 3.4. Government Subvention Support

Since 2011–12, AFCD has allocated approximately HK$1 million annually to support animal welfare organisations. Supported activities include re-homing centres, desexing, education, and facility improvements [9]. In the last 5 years, the total annual actual subvention amount released to organisations who bid for it has consistently exceeded $1,000,000, with an average of $1,353,161 in each financial year since 2020. The increase in funding is reflected in the increase in applications, with 11–15 applications made each year between 2019 and 2025. The majority of applications were submitted either for operational purposes or with operational needs included among their stated objectives. However the AFCD has stated that applications to support recurrent expenses such as monthly rent, utility bills and staff salary are deemed not normally appropriate for subvention support [10].

As noted above, most interviewees complained that lack of regulation of online fundraising was diverting funds from long-established operators. They suggested that given the uneven donation landscape and lack of oversight caused, the government should provide more significant subvention support to shelters promoting the government’s welfare policies.

*Our subvention is mainly used for sterilization and adoption, transportation. A small part is training and public education for example an adoption event, talks. These are usually the four areas. But the subvention won’t do much. Neutering a dog costs HK$3000 to $4000. We’re not adopting out pomeranians or poodles! The desexing costs for a dog weighing over 20 kg are twice as much as for a dog weighing less than 10 kg. We actually rely mostly on individual monthly donations from the public.*

SS 06

*Government could give us an allowance to rent a place for the number of animals we keep. The more animals you have, the bigger the place to rent, the more expensive. To be enough, [the allowance] needs to be reasonably high. The other thing is medical care. We have actually applied to the AFCD for some funds for medical care. But it is not very much.*

SO 05

For long-term sustainability, relying on fundraising as the sole source of income for shelters is problematic, as donations are an unstable source of income and do not allow for certainty in financial planning. In the current economic environment government support is perceived as crucial. All interviewees complained that the support provided by the government’s annual subvention to shelters is inadequate despite government records showing an increasing trend over the past 15 years.

### 3.5. Government Built Shelters

The Development Bureau has provided some funding to two shelters to build facilities on non-occupied government land identified by the Lands Department as suitable for short term tenancies at nominal rent. These shelters are required to take a certain number of animals from AFCD in exchange for the support. Some of the animals involved had to be abandoned by their owners due to the government’s development projects which closed villages in the northeast New Territories.

Some operators were supportive of this initiative:

*I think the government could actually provide more resources. For example, in the previous land acquisition plan in the Northeast, they actually allocated some wasteland and abandoned land to certain organizations. The government then provided funds to build shelters, and then offered these shelters at a very low, symbolic rent. This is a mutually beneficial approach. Land acquisition has resulted in many village dogs being abandoned. Because you acquire land, your indigenous residents will be provided with public housing by the government. However, many dogs and other animals are unable to follow their owners into public housing. When the owners have no choice, they can only give up their pets. When the government provides some resources, those households can use these associations to help them take in these animals.*

SS 01

There were many, however, who suggested that the support given to these shelters had enabled the government to pass on its responsibilities for effective animal management policies.

*The Development Bureau allocating tens of millions to build a shelter that can only house a hundred or a few dozen dogs is very inefficient. Conversely, we suggest that for TNR dogs the government should provide subsidies of HK$1000 each. Partnering with some veterinary clinics. for every dog or cat captured, you pay the clinic 1000 dollars to handle the treatment. Although the clinic doesn’t make much profit, it helps balance costs and benefits the animals. The government can also save a lot of money. It’s impractical to spend tens of millions each time to build shelters that can only house a few dozen dogs. Conversely, with 1000 dollars per dog, the clinic can balance costs and help the dogs and cats, reducing their numbers. The government spends less money.*

SO 04

*Kwu Tong was going to be developed into a new city centre, so they needed to take back the land. At that time, there was public outcry because a lot of people were squatters and they all kept cats for the mice, because of burglars, they will keep dogs. So there were about 3000 animals that would become homeless due to the government taking back the land. People made a big fuss and kept making noise. So the government said: “OK I am doing a huge project, several billions [dollars], but you all are making such a fuss about a few thousand animals. You are stopping me from developing.” So the government paid out money and the Development Bureau built shelters for some organizations and delegated the problem to them. I feel like, those organisations are banding with the government to oppose us on speaking the truth about the need for TNR so they can be friendly with the government. I won’t do things like this.*

S0 03

## 4. Discussion

Munro has proposed that a social constructivist approach may be instructive in gaining a deeper understanding of the animal protection movement [11]. Munro illustrates how individuals become activists after engaging in ‘social problems work’ with others. Members of social movements, such as the animal protection movement, commit to their cause through their shared collective identities [12]. From this perspective, animal protection is a collective pursuit, socially constructed by interaction between advocates and critics, that frames the problem of unwanted animals as a social problem to be addressed by the public.

In Hong Kong, animal shelters have emerged as a new type of institution to challenge entrenched government practices of euthanasia in government kennels as the solution to unwanted animals. In 1988, DiMaggio coined the term ‘institutional entrepreneurs’ [13] to describe new actors who seek political opportunities to frame issues and problems, to collectively drive new beliefs, norms and values into social structures [14]. In this sense, animal shelters have been at the forefront of driving narratives to mobilise public support against euthanasia as an animal management policy.

Schuab et al. [15] conceptualise moralization as a narrative strategy in which policy actors argue for or against policy proposals based on moral values. Using the Narrative Policy Framework, they studied the moralization in German policy debates over climate change and public health. The Narrative Policy Framework posits that narrative strategies are used by actors to influence policy making. Strategies comprise four elements; a *specific policy setting*, *specific narrative characters* used by actors, *a certain policy solution* advocated as the ‘happily ever after’ or moral of the story, and finally, a *plot* that makes sense of the setting and its characters in relation to the proposed policy solution.

Narrative characters take four archetypes; *villains*, who are blamed for the policy problem or the failure to find solutions to it; *victims*, who suffer the negative consequence and *heroes*, who save the day by solving or preventing policy problems for certain *beneficiaries* [16]. Utilising this framework, we suggest the government’s use of euthanasia to address unwanted animals in its kennels has been established as a specific policy setting, with several narrators. The government is seen as the *villain*, responsible for being indifferent to animal suffering and persisting in a flawed policy of euthanasia where animals, as *victims,* have suffered harm. Animal shelters may position themselves as the *heroes*, working towards solving the problem through rehoming abandoned animals from the government as well as carrying out desexing programmes that target the root of animal overpopulation. The plot is to end the unnecessary loss of animal life.

Without a strong narrative rooted in research or animal welfare science, debates about animal welfare have largely remained moral ones in Hong Kong. The AFCD is portrayed as an uncaring, ruthless, or apathetic villain despite having the power and resources to change an unpopular status quo through a humane population control policy, such as TNR. Within this framework, animal shelters, who were previously cast as heroes who save animals lives, are now being challenged by other actors who compete for the same resources and legitimacy. Under the onslaught of competition from those who are ‘selling tragedy’, shelters have begun to lose their *hero* status as under-resourced underdogs, even though they may struggle financially. Where they find ways to generate income and professionalise their operations, shelters may be perceived to have gained political influence and resources, reducing their need for public support. Government assistance to these organisations lends credence to a growing narrative that animal shelters that are aligned with the government, such as those built with Development Bureau funding support, have compromised their morality and are unwilling to challenge the status quo.

Interviewees have cautioned that even animals in established shelters are not spared. They are no longer the *victims* in a changing narrative. Animal shelters risk losing their status as heroes where they refuse to ‘sell tragedy’ and choose to limit their intake of animals to manageable numbers, recognising that they need to maintain reasonable animal welfare standards for their existing charges.

Animal rescue groups have now emerged to fill the shoes of the underdog hero, where the victim is now an animal which requires urgent and substantial medical treatment. The public have the opportunity to align with this new hero by providing money to save the animal utilised to ‘sell tragedy’. Ironically, this narrative provides an incentive for animals to be kept in states of extremely poor welfare for the sake of fundraising and undermines welfare improvements in established animal shelters by diverting limited resources away from the group to single animals in critical condition that may be suffering immensely.

Appeals to emotions play a key part in motivating action in social movements [17]. Social media is a popular way for those in the animal protection movement to share their values and promote collective action [18]. The advent of social media as a means to fundraise online has allowed those players in the animal movement to connect with hundreds and even thousands of individuals, levering, for financial gain, emotional connections over a shared concern [19].

Interviewees in our study identified a developing strategy employed by some involved in sheltering and rescuing to ‘sell tragedy’ to the public via social media posts of animals in urgent need of veterinary care as a means to fundraise. Interviewees complained that emotional posts of individual animals were being used unethically to motivate donations for crisis over sustainability.

The concerns identified by interviewees are supported by behavioural studies. Identifying a needy beneficiary is a known motivator for acquiring very large numbers of small charitable donations [20]. Providing information about an identified individual is more likely to tap into donator’s sympathies than references to a large number of unidentified beneficiaries in need [21].

Giving is also a known contributor to a positive self-image as an altruistic and empathetic person [22]. In Hong Kong, social media posts for injured animals seeking donations often show comments from donors identifying themselves as having sent money and sharing screenshots of their donations.

In addition to the difficulties shelters face in securing adequate public donations to ensure sustainability, rather than avert crisis, interviewees questioned whether the AFCD’s current policies for promoting animal welfare are addressing critical components of the problem: the number of animals that enter shelters and remain there long-term and the lack of humane stray animal population control. Shelter operators complained that it is too easy for members of the public to abandon pets, even dogs with microchips, without legal consequences. Their observations have a sound basis. Between 2020 and 2025 there were no prosecutions for animal abandonment [23].

A significant concern amongst our interviewees was that AFCD’s policy to pass animals to shelters had shifted the problem of unwanted animals to those least empowered to solve it. In the current context of overcrowded shelters, struggling for sufficient resources to care for the animals they have, effective policies to stem the flow of unwanted animals are needed as much as, if not more than, increased financial support. AFCD’s policies around dealing with unwanted animals were identified as largely ineffective. Along with the failure to prosecute owners for abandonment, it was noted that the current licencing scheme does not reward those who chose to desex their pets though lower licencing fees. In the context of serious health concerns with smuggled animals entering Hong Kong from China, where rabies remains a problem, it would also seem sensible to provide incentives to owners to vaccinate their dogs.

While government strategies around achieving responsible pet ownership require revision, it is relevant to note that in recent years the AFCD has made a significant U-turn in its policy on animal management. Under considerable pressure from the public, the AFCD has drastically reduced the number of stray animals collected by animal catching teams and euthanised. The total number of dogs and cats euthanised in government kennels dropped from 10,467 in 2010 to 205 in 2025 [24]. For dogs and cats with suitable temperament collected by AFCD, the adjusted policy is now to try to actively rehome them through private shelters or individuals. The Department has partnerships with seventeen shelters to which they provide animals for adoption. These animals are offered from the government kennels to the shelters.

One shelter supporter explained:

*AFCD don’t offer an adoption service. Instead, they distribute them to their partners like us, called Animal Welfare Organisations. They will ask us if we’ll accept these cats and dogs. We primarily accept referrals from them. There is also more than one centre. Each district makes requests to us. They operate independently so, I might get inquiries from three or four [AFCD] centres a month asking if we’ll take their cats and dogs.*

SS 16

While many interviewees observed that AFCD has failed to humanely manage the unowned dog population and shifted the burden of unwanted animals onto shelters, reducing stray catching rates and consequently euthanasia is a significant policy change and warrants discussion. Since its inception AFCD has maintained that catching stray animals was a necessary intervention for population control and rabies containment [25]. However no rabies cases have been reported in Hong Kong since the 1980s and considerable public criticism was made of the policy for decades [26]. The updated policy to reduce the numbers of animals caught and to allow for rehoming through shelters taking animals from AFCD is supplemented by the government’s decision to assist in the building of two new shelters in the New Territories to help take animals left homeless by the Northern Expansion.

In taking into account significant public dissatisfaction to change a longstanding policy, the AFCD has shown a willingness to work with stakeholders to align their mission with public opinion. Decades ago, this would not have seemed possible, but this change fits within the dynamics of policy making in a changing world. In established theories of policy analysis, it is appropriate that objective science should be utilised to form rational and legitimate policies addressing societal problems [27]. However, in recent years, political scientists have noted that the legitimacy and efficacy of previous institutionalised policies have become increasingly susceptible to challenges by new perceptions of what is acceptable [28]. As Hong Kong society has become increasingly concerned with animal protection, established policies for animal management have come under challenge [29].

The new order for policy making recognises that societal actors have a significant role in evaluating and driving change [28]. The authority of positions based wholly on scientific expertise are increasingly questioned and challenged by the public based on their own personal experience [30]. A pertinent example of such a challenge in practice is evidenced by the widespread rejection by interviewees of the government’s position that a TNR trial in 2015 for two districts did not deliver the results necessary for a territory-wide government-led TNR policy. Interviewees in this study unanimously questioned the legitimacy of the government’s position on TNR as an appropriate means to manage the stray/feral population of dogs in Hong Kong.

In the new context of cultural politics [31] the old method of determining policy has been destabilised, along with trust in the government to deliver efficient and legitimate solutions. Against this background, it has become increasingly necessary for policy makers to build trust with stakeholders, as support in institutional processes cannot be assumed [30]. A study in Hong Kong found opportunities for citizens to participate in and affect the development of health care policy increased public trust in the health care system and in the government [32]. To increase opportunities to build trust it is critical for policy makers to understand what the perceptions of stakeholders are and engage with evolving societal views.

## 5. Implications for Policy and Legislation

### 5.1. Regulating Fundraising

In Hong Kong, the government has pledged to investigate fundraising activities which are suspected to involve money laundering, fraud and other unlawful acts [33]. However in the absence of complaints of fraud, they will not intervene to scrutinise or oversee the posting and fundraising practices of animal welfare organisations online. A concern outside of the purview of the AFCD but relevant to the Social Welfare Department is the lack of regulation around fundraising, which gives the green light for people to fundraise for whatever animal ideal they promote, including crisis, without scrutiny of where the money goes. This lack of oversight appears to have inadvertently fostered an environment for animal cruelty to occur by encouraging suffering. To combat these concerns, it is recommended that the Social Welfare Department act to control online fundraising activities by requiring registration as charities before donations can be sought. Those interviewees holding charity licences believe the audit progress under the Inland Revenue Department is rigorous and the best way to hold organisations accountable. However, as online fundraising is easy, even for legitimate shelter operators, the incentive not to register as a charity persists. In 2020, a report by the Hong Kong government Legislative Council’s Research Office reported the need for Hong Kong to enact a dedicated Charities Law providing oversight of those taking donations from the public [34]. Lack of regulation in this area should now be addressed.

Lack of regulation on fundraising may have significant social repercussions, including providing the opportunity for fraud. Studies in the USA have found that most people supporting charities in America do so without investigating the effectiveness of the organisation first [35]. Without sufficient oversight, ineffective use of funds may result from both hard and soft corruption. Hard corruption occurs when charity funds are misappropriated or funnelled inappropriately to the management. Soft corruption involves use of funds that is not corrupt per se, but which may be legitimately questioned by donors (use of donations to support unreasonable management costs and fundraising expenses).

In 2025, four animal shelters in Hong Kong discovered that their names and good will with donors were being used by fake shelter websites to seek donations from the public [36]. These acts of fraud were reported to the Hong Kong police and are prosecutable under the criminal laws of Hong Kong.

In 2024, to highlight the prevalence of fraudulent content posted by persons masquerading as animal rescue groups, the Social Media Animal Cruelty Coalition (part of the Asia for Animals Coalition) documented 1022 links over 6 weeks to suspected fake posts across five social media platforms. In total, 30% of posts showed false abandonment images and one fifth of all posts were seeking donations from the public [37].

Fraudulent posts used to secure donations through social media platforms are a growing international concern. Another large-scale study conducted in 2024 of 3 million posts for donations across five social media platforms found 832 scams cases. Of these, emotional pleas for money to support urgent care for animals was identified as one of the top ten most common scam typologies, with 125 of 679 posts identified by the authors of the study as scams [38].

Even where posts are not fraudulent, transparency in online donation use is critical to maintain public trust. In order to better control online fundraising, some countries have seen fit to introduce new laws and re-interpret current laws to manage its growing popularity. In Finland, the Money Collection Act 863/2019 requires all solicitations of money from the public for a philanthropic purpose to be registered with the National Police Board and recipients must provide an account of the use of donations received. Similar requirements are imposed in Denmark through the 2014 Danish Collection Act, in Iceland under the 1977 Act on Public Fundraising and in the Philippines via the 1978 Solicitation Permit Law. While the Icelandic and Philippine fundraising laws both preceded the internet, they have since been clarified by authorities to include regulation of online donations.

### 5.2. Promoting Animal Welfare Standards

Shelters provide support to the government by housing abandoned and unwanted animals. However, they do so without guidance from the government as to appropriate welfare standards. The AFCD guidelines for organisations applying for subventions state that only those applications that are project-based and directly related to the promotion of animal welfare and proper animal management will be considered. Examples provided by the Department of programmes that have received subvention funding in the past include programmes to enhance public awareness of responsible pet ownership, animal welfare and adoption; programmes to assist AWOs in running rehoming schemes, such as provision of preventive veterinary medical care (including desexing) and food for rehoming animals; and programmes to renovate or enhance facilities/equipment in an adoption centres.

While education on responsible pet ownership is more likely to be funded than other subvention proposals, the Department’s objectives around achieving animal welfare outside of responsible pet ownership remain unclear. In this context it is critical for the government to ensure their education and behavioural messaging and the proposals they fund by subventions are actively supporting responsible pet ownership by meeting their objectives. In the absence of a clear animal welfare policy, decisions around what to fund and determinations of whether the proposals funded have helped to achieve policy goals remain opaque and open to challenge.

Similarly to Hong Kong, lack of regulation on appropriate care standards in the USA has caused problems for the welfare of animals in shelters, with cases of animal cruelty resulting from failures to recognise or adequately respond to suffering [39]. Only a small number of States have passed legislation specifically to licence shelters but the Association of Shelter Veterinarians have developed guidelines for standards of care for animals in shelters in North America, albeit without legislative teeth [40]. The most recent update to the guidelines in 2022 focuses on ensuring the welfare of animals through recognition of the Five Domains model.

In the UK, the Animal Health and Welfare (Scotland) Act 2006 applies to animals in shelters, requiring carers to meet the animals’ welfare needs in accordance with the Five Freedoms. However, despite the law, cases of animal neglect and misuse of public donations by shelters have prompted the passing of specific legislation to require shelters to be licenced, allowing for inspections of facilities to ensure the welfare of the animals therein [41]. The UK government is also currently considering the licencing of shelters under the 2025 Animal Welfare Strategy for England. Introducing similar regulatory controls for standards of care in shelters in Hong Kong would provide an appropriate framework for the AFCD to guide shelters to improve animal welfare.

A related issue, requiring the introduction of clear policy leadership from AFCD, concerns the use of suffering animals, with no scope for an improved quality of life, as a means to attract funds. For this purpose, interviewees complained that animals may be kept alive to attract donors, when there are legitimate questions around quality of life, such as when an animal has been paralysed and will live its life in a shelter. While emotional beliefs about the importance of animal protection provide a legitimate basis on which a shelter may connect with the public, continual posting of suffering animals to fundraise is not a sustainable or ethical model for financial stability. For those shelters relying on a continual ‘crisis’ model for operational costs, a constant supply of sick or injured animals in need of veterinary care is necessary. If an animal dies or is no longer a sufficiently tragic case, a new animal will need to be identified, to take its place, in order to ensure the funding supply continues. Animal cruelty is prohibited in Hong Kong but no duty of care provisions have been legislated to protect the wellbeing of animals, other than those used for commercial purposes (dogs, cats and reptiles in trade and dogs in breeding establishments). Duty of care law is important as it sets minimum standards for the care of animals to protect their welfare. In the absence of a requirement to meet positive animal welfare standards it is possible to ignore the need to end unnecessary suffering. While a 2019 public consultation proposing that those who keep animals should provide them with a duty of care received support from 88% of respondents [42], lack of subsequent action to enact duty of care legislation and lack of public education on what duty of care entails has allowed those seeking funding from the public to set their own standards. In the absence of education, duty of care has been interpreted by some to mean the duty to keep animals alive, no matter what. Without education on how to identify animal welfare, and legislation which requires people to provide animals with a reasonable quality of life, the threshold of when suffering has moved to become unacceptable may be unclear to the public and is open to exploitation for fundraising purposes.

In a number of jurisdictions across the world, including the UK, Australia, the EU, Taiwan and Singapore, it has been recognised that the most effective way to prevent animal abuse is to impose a duty of care on those who keep animals, requiring them to provide their animals with a reasonable standard of care. Imposing a proactive duty of care ensures that animals need not suffer unnecessarily before action can be taken to protect them from harm. The duty of care imposes legal responsibility on those caring for animals to ensure the conditions in which they are kept meet positive welfare standards. Were duty of care legislation passed in Hong Kong, authorities would be empowered to hold those prolonging the poor welfare of animals, to drive up fundraising, accountable and educate the public to better recognize and condemn failures to provide shelter animals with appropriate care.

### 5.3. Improving Animal Management Policies

From 2000 onwards, the AFCD has set a KPI of 80% vaccination of dogs for rabies [43]. Rabies vaccinations in Hong Kong are tied to applications for dog licences registering ownership. All owners of dogs over 5 months of age must register their ownership and have their dogs vaccinated for rabies [44]. AFCD Departmental Reports between 1981 and 1997 show that vaccination rates for dogs were 30,000 to 40,000 annually. In 2025, the Secretary for the Environment and Ecology stated that 158,663 dogs (of an estimated 221,000) were licenced as vaccinated in Hong Kong in 2024 [45].

An upward trend in licencing and vaccination of dogs may well be regarded as an indication of increasing acceptance within the community of responsible pet ownership. However, with all interviewees stating that their shelters are at full capacity, adoption rates are low and there are more animals in need than they have the resources to support; it appears the implementation of the government objective around responsible pet ownership needs to be re-adjusted to fit current realities. All interviewees observed that the current policy around animal management is not effective and change is necessary. A government-led TNR programme to reduce the population of free roaming dogs is perceived by all interviewees as a necessary and effective step towards improved animal welfare.

Endorsed by the World Organisation for Animal Health, the Alliance for Rabies Control (UK), Humane World for Animals (formerly Humane Society International), RSPCA International and World Animal Protection, TNR programmes are widely recognised internationally as providing a humane method for stabilising dog populations and controlling rabies. TNR programmes are used to manage unowned dog populations in Australia, the USA, Canada, India, Europe, parts of Africa, South America and the UAE. Data from the WHO indicates that culling produces only a short-term effect and even at maximum catching rates, euthanasia has no significant impact [46]. TNR, combined with mandatory microchipping and deterrent abandonment laws, has significantly reduced the number of stray dogs in the Netherlands [47]. In Taiwan, overcrowded shelters led to a policy to use TNR programmes, run by NGOs with government support, to control dog populations [48]. The success of both euthanasia and TNR to control dog populations depends largely on abandonment rates [49] making the prosecution of those who abandon their dogs a priority.

The stated mission of the Quarantine and Inspection Division of the AFCD is to promote animal welfare and stray animal management [50]. Meeting this mission requires the government to address the problem of animal abandonment which continues to impact animal welfare and the number of stray animals in Hong Kong. Shelters provide support to the government by housing abandoned and unwanted animals. However, they do so without guidance from government as to appropriate welfare standards. While the promotion of responsible pet ownership is a pathway to reducing animal abandonment, the Department has not publicly identified the KPIs necessary to improve animal welfare in Hong Kong. It is recommended that these are identified to assist shelters to align with the KPI’s, improving their chances of AFCD support by increased subventions. In the absence of a clear animal welfare policy, decisions around what to fund and determinations of whether the proposals funded have helped to achieve policy goals remain opaque and open to challenge.

## 6. Conclusions

This study was conducted in Hong Kong and its findings are therefore limited to that jurisdiction. The sample size was relatively small but included approximately 50% of shelter operators, within which a consensus in views was identified. Overwhelmed shelters in Hong Kong identified inadequate regulatory oversight, insufficient funding support and a failure by the government to promote animal welfare standards as presenting serious impediments to the well-being of shelter animals. Despite purporting to support the concept, the AFCD’s objectives around achieving animal welfare, outside of responsible pet ownership, remain unclear. Passing on animals collected by the government to already overburdened shelters does not improve animal welfare. In the absence of coherent and meaningful government policies on animal welfare, the animal protection community have arguably become the primary bearer of responsibility for unwanted animals and the authority on acceptable animal welfare practices, allowing for significant variations of standards.

Interviewees were of the opinion that the AFCD’s failure to implement humane population control policies has resulted in the unsustainable numbers of animals now being placed in shelters. Previously, adoption practices by the public had helped relieve the pressure to some extent, but many of those who could adopt no longer are able to do so and shelters are full. The subventions provided are insufficient to provide the support necessary to the numbers of animals, particularly dogs, now living in shelters long term.

It is a classical ambition of legal regulation to change behaviours. Behavioural changes are most efficiently achieved when attitudes about the morality of the behaviours change, leading to self-regulation [51]. The purpose of animal protection legislation is to give accepted moral norms a legal status and to educate the public to conform to those norms, either through fear of sanction or because they have internalised the need to do so [30]. As the government department responsible for the promotion of animal welfare in Hong Kong, it is incumbent on the AFCD to lead public education on accepted standards of care and promote adoption of those standards in unwanted animal management, including in shelters. In the absence of education on acceptable standards of care for animals, the public has no guidance on what to support and those who seek to exploit animals for profit are free to ‘sell tragedy’, detracting funds from shelters already in financial need and undermining animal welfare.

## Figures and Tables

**Table 1:** Background of interviewees.

					Years of Experience		
Role		Total	1–5 years	6–10 years	11–15 years	16–20 years	Over 20 years
Shelter Operator	SO	10	0	6	0	0	4
Manager/Support	SS	12	1	1	5	1	4

## Data Availability

The data supporting reported results is not made available due to privacy concerns.

## Supplementary Materials

The following supporting information can be downloaded at: https://www.mdpi.com/article/10.3390/ani16192994/s1, Table S1: Shelter owner questionnaire.
