# Supplementary material for: An Empirical Study of the Operational Challenges for Private Animal Shelters in Hong Kong and Their Links to Regulatory and Policy Gaps
Source: Animals (Basel). 2026 Sep 23;16(19):2994. doi: 10.3390/ani16192994 (PMC13663606; doi:10.3390/ani16192994)
Supplement: Supplementary file 1 [file animals-16-02994-s001.zip › animals-4415121-supplementary.pdf]

Table S1. Shelter owner questionnaire

	Questions	Probe further
Personal experience prior to working in shelters	Can you tell me about your working background prior to being a shelter owner?	
Experience with animal related networks	Were you involved in any animal related work or volunteering?<br><br>How long were you doing that before you opened your shelter?<br><br>Had you placed any animals with shelters before opening your own shelter?	Are you connected to any animal associations?<br><br>Have you rescued any animals?<br><br>[If Yes] How many and what type of animals? Can you tell me why you placed those animals in that shelter?
Background and profile of animal shelter	Can you tell me about the background of your shelter?<br><br>Why did you open a shelter?<br><br>What services does your shelter offer? (e.g. boarding, grooming, adoption, surrender etc.)<br><br>What are the goals of your shelter? (e.g. adoption, boarding, sanctuary)<br><br>What were/are the challenges setting up and running your shelter?	
Sources, profile and ownership status of animals	Where do your animals come from?<br><br>Why do people bring animals to your shelter?<br><br>What is the profile of people who use your shelter? (e.g. volunteers, owners requiring boarding services)<br><br>Does your shelter have any specific criteria regarding accepting animals?<br><br>Are most of the animals in the shelter registered to you, as owner?<br><br>Under what circumstances are animals not registered under your name?	How do people find out about your shelter?
Operations and staffing	How many animals and what type of animals does your shelter have?<br><br>How large is your shelter? (space)<br><br>Do you have any help running your shelter?	

	Do you have any employees working in your shelter? If yes, how many?<br><br>Do you have any volunteers working in your shelter? If yes, how many?<br><br>What are their main duties hired staff to help with?<br><br>What are the major challenges you face as a shelter owner?	
Finances	Is your shelter profit making or non-profit making?<br><br>Could you tell us how you financed setting up the shelter?<br><br>Could you share the cost of running a shelter e.g. rent, food, manpower costs?<br><br>How are your costs covered? Can you share with us how you fund raise?<br><br>Do you receive regular payments or donations? What form do these donations usually take?<br><br>How can donors/potential donors track how donations money is spent?	Are you a registered business or charity?<br><br>[If unwilling] Could you breakdown the expenditure by percentage?<br><br>Are there any charges for placing an animal in your shelter?<br><br>Is your shelter financially transparent?
Knowledge and opinion of the shelter landscape in Hong Kong	How would you describe the demand for animal sheltering?<br><br>In your opinion, what contributes to that demand?<br><br>According to your knowledge, how do most animal shelters operate?<br><br>How do you assess the overall practice of animal sheltering in Hong Kong?	Can the shelters be divided into different types? e.g. financing (profit vs non-profit), fund raising receiving animals, hiring staff, daily operation, accessibility by the public, etc.?<br><br>Do you think animal shelters are necessary?<br><br>Do you think the standard of animal shelters is adequate?

Knowledge and opinions on “bad shelters” and penalties under the law	**Stray Wonderland**<br>On 29 April 2019, an animal shelter, "Stray Wonderland", was prosecuted for cruelty to animals. Police found 28/102 dogs and 8/44 cats housed on the premises had died of neglect, and the rest were in poor condition. Do you know about this incident?<br><br>What do you think were the issues that led to that situation?	
Opinions on legislation and regulation	In your opinion, what makes a good shelter?<br><br>In your opinion, what makes a bad shelter?<br><br>What actions, if any should be taken to combat bad shelters?	
Specific regulation	Do you think there is any specific legislation that can be used to help shelter owners?<br><br>Should there be minimum standards for animal shelters?<br><br>In your opinion, do you think there should be any training or licensing requirements for people who want to be shelter owners?<br><br>Do you think shelters should be required to make public their donations by law?<br><br>In your opinion, how would these regulations affect animal sheltering?<br><br>Do you foresee any obstacles/ challenges in regulating shelters?	Could you tell me more what recommendations you have e.g. in terms of manpower, space, facilities, husbandry, vet treatment, food & water?
	Do you have any vision for the future of your own or other shelters in Hong Kong?<br><br>Do you have any suggestion as to who else we should speak to regarding this issue?	
